# Five-year results of a treatment program for chronic hepatitis B in Ethiopia

**DOI:** 10.1186/s12916-023-03082-4

**Published:** 2023-09-29

**Authors:** Hailemichael Desalegn, Stian Magnus Staurung Orlien, Hanna Aberra, Eyerusalem Mamo, Sine Grude, Kristina Hommersand, Nega Berhe, Svein Gunnar Gundersen, Asgeir Johannessen

**Affiliations:** 1 Medical Department, St. Paul’s Hospital Millennium Medical College, Addis Ababa, Ethiopia; 2 Department of Infectious Diseases, Vestfold Hospital Trust, Tønsberg, Norway; 3 Department of Paediatrics, Vestfold Hospital Trust, Tønsberg, Norway; 4 Regional Advisory Unit for Imported and Tropical Diseases, Oslo University Hospital Ullevål, Oslo, Norway; 5 Faculty of Medicine, University of Oslo, Oslo, Norway; 6 Aklilu Lemma Institute of Pathobiology, Addis Ababa University, Addis Ababa, Ethiopia; 7 Department of Global Development and Planning, University of Agder, Kristiansand, Norway; 8 Institute of Clinical Medicine, University of Oslo, Oslo, Norway

**Keywords:** Hepatitis B virus, Antiviral treatment, Survival analysis, Resource-limited settings, Longitudinal cohort study

## Abstract

**Background:**

In sub-Saharan Africa, less than 1% of treatment-eligible chronic hepatitis B (CHB) patients receive antiviral therapy. Experiences from local CHB programs are needed to inform treatment guidelines and policies on the continent. Here, we present 5-year results from one of the first large-scale CHB treatment programs in sub-Saharan Africa.

**Methods:**

Adults with CHB were enrolled in a pilot treatment program in Addis Ababa, Ethiopia, in 2015. Liver enzymes, viral markers, and transient elastography were assessed at baseline and thereafter at 6-month intervals. Tenofovir disoproxil fumarate was initiated based on the European Association for the Study of the Liver (EASL) criteria, with some modifications. Survival analysis was performed using the Kaplan–Meier method.

**Results:**

In total, 1303 patients were included in the program, of whom 291 (22.3%) started antiviral therapy within the initial 5 years of follow-up. Among patients on treatment, estimated 5-year hepatocellular carcinoma-free survival was 99.0% in patients without cirrhosis at baseline, compared to 88.8% in patients with compensated cirrhosis, and 54.2% in patients with decompensated cirrhosis (*p* < 0.001). The risk of death was significantly higher in patients with decompensated cirrhosis at baseline (adjusted hazard ratio 44.6, 95% confidence interval 6.1–328.1) and in patients older than 40 years (adjusted hazard ratio 3.7, 95% confidence interval 1.6–8.5). Liver stiffness declined significantly after treatment initiation; the median change from baseline after 1, 3, and 5 years of treatment was − 4.0 kPa, − 5.2 kPa, and − 5.6 kPa, respectively.

**Conclusions:**

This pilot program demonstrates the long-term benefits of CHB therapy in a resource-limited setting. The high mortality in patients with cirrhosis underscores the need for earlier detection of CHB and timely initiation of antiviral treatment in sub-Saharan Africa.

**Trial registration:**

The study was registered at ClinicalTrials.gov (NCT02344498) on January 26, 2015.

**Supplementary Information:**

The online version contains supplementary material available at 10.1186/s12916-023-03082-4.

## Background

Chronic infection with hepatitis B virus (HBV) is a major global health threat with an estimated 296 million people living with chronic HBV infection worldwide [1]. In the absence of treatment, 15–40% of individuals with chronic hepatitis B (CHB) will eventually die from the disease, due to liver failure, cirrhosis, and/or hepatocellular carcinoma (HCC) [2]. In 2019, approximately 1.5 million individuals acquired new HBV infections, with a corresponding 820,000 annual deaths attributable to HBV-related complications [3]. In 2016, the World Health Organization (WHO) endorsed a plan to eliminate viral hepatitis as a public health problem by 2030 [4]. To reach this target, a number of interventions are needed, including universal screening and access to antiviral treatment.

Antiviral treatment reduces the riskof cirrhosis and HCC in those living with CHB [5–7]. Despite the global high prevalence and mortality, however, estimates show that only 5% of treatment-eligible patients receive antiviral therapy [8]. Long-term studies from high-income countries have shown that tenofovir disoproxil fumarate (TDF), a nucleotide analogue and inhibitor of HBV polymerase, is efficacious, safe, well-tolerated, and has a high barrier to resistance [7, 9–13]. The price of generic TDF dropped to US$30 per person per year with the expiry of patent protection in 2018, and thus the drug cost is no longer the main limiting factor [3]. Still, only a fraction of patients with CHB in sub-Saharan Africa have access to antiviral therapy. Barriers include lack of funding, complex treatment guidelines, regulatory restrictions on antiviral drugs, and limited access to laboratory tests and fibrosis assessment tools [14].

There is hardly any data on the long-term effects of antiviral treatment of CHB in sub-Saharan Africa. The WHO has recognized this as an important knowledge gap and called for longitudinal cohort studies [15]. Herein, we present 5-year results from one of the first large-scale CHB treatment programs on the continent. Our study sheds light on the feasibility and efficacy of CHB treatment in this setting, which is key to planning future interventions to eliminate viral hepatitis as a public health threat by 2030.

## Methods

### Study setting and participants

Adults (≥ 18 years) with CHB were recruited between February 9 and December 14, 2015, at St. Paul’s Hospital Millennium Medical College, a referral hospital in Addis Ababa, Ethiopia. CHB was defined as being seropositive for hepatitis B surface antigen (HBsAg) for more than 6 months. At the time the program started, there were no public-sector treatment programs in Ethiopia for mono-infected HBV patients, although a small number of patients were able to import drugs from abroad or buy from inconsistent ‘black markets’. A detailed description of the study population, design, and patient assessment has been reported previously [16, 17].

### Treatment decision and follow-up procedures

There are no African HBV treatment guidelines; thus, the treatment eligibility criteria were based on the European Association for the Study of the Liver (EASL) Guidelines published in 2012 [18], with a few modifications. Liver biopsy was unrealistic in this setting and was replaced with transient elastography (Fibroscan® 402, Echosens, France). Based on a previous meta-analysis and a study from West Africa [19, 20], we used a Fibroscan threshold of 7.9 kPa to define significant fibrosis and 9.9 kPa to define cirrhosis.

The two EASL criteria pertaining to liver inflammation (Metavir ≥ A2 with viral load > 2000 IU/ml, and alanine aminotransferase (ALT) > 80 IU/L with viral load > 20,000 IU/ml) were merged into one: ALT > 80 IU/L with viral load > 2000 IU/ml. Furthermore, since African patients might have an increased risk of developing HCC [21], a new eligibility criterion was added, namely, HCC in first-degree relatives and elevated viral load.

Hence, patients who fulfilled the following criteria were considered eligible for treatment:

1. Decompensated cirrhosis
2. Compensated cirrhosis (confirmed with ultrasound and/or transient elastography > 9.9 kPa)
3. Significant fibrosis (transient elastography > 7.9 kPa) and viral load > 2000 IU/mL
4. Moderate/severe liver inflammation (ALT > 80 IU/L) and viral load > 2000 IU/mL
5. HCC among first-degree relatives and viral load > 2000 IU/mL

Decompensated cirrhosis was diagnosed if there was past or present evidence of ascites (diagnosed clinically and/or by ultrasound), hepatic encephalopathy, bleeding oesophageal varices, or jaundice.

Patients with known or suspected HCC were not included in the study but referred to further diagnostic work-up and/or therapeutic management. Likewise, those who were found to be HIV positive at baseline or during the course of the study were excluded and transferred to the local HIV treatment unit.

Information on previous and current alcohol intake was obtained at baseline using a frequency/quantity questionnaire. Daily alcohol consumption of > 20 g in women and > 30 g in men, for a minimum period of 6 months, was classified as alcohol misuse.

Co-infection with hepatitis C virus (HCV) was detected at baseline using an enzyme-linked immunosorbent assay (ELISA) method measuring anti-HCV in serum (Elisys Uno, HUMAN, Wiesbaden, Germany), whereas antibodies to hepatitis D virus (anti-HDV) was detected by ELISA in plasma samples (ETI-AB-DELTAK-2, Diasorin, Italy) and intermediate or weak positive results were confirmed by a second anti-HDV ELISA (Dia.Pro Diagnostic Bioprobes Srl, Milan, Italy). Treatment of HCV and HDV co-infections was not available through this program.

Antiviral treatment for hepatitis B (TDF), laboratory tests and transient elastography, was provided free of charge throughout the study period.

### Follow-up of patients eligible for antiviral treatment

Before starting antiviral treatment, eligible patients were obliged to complete adherence counselling. Tenofovir disoproxil fumarate (TDF) (Viread®, Gilead Sciences, Inc., Foster City, CA, USA) 300 mg once daily was prescribed to patients fulfilling the treatment eligibility criteria given above.

After initiation of antiviral treatment, patients were followed up after 2 and 4 weeks, thereafter at 3-month intervals. The follow-up appointments focused on treatment efficacy, adverse drug effects, adherence monitoring, and laboratory tests, including standard biochemistry (Humalyzer 3000, Human, Germany) and haematology (Huma-Count 30, Human, Germany). Screening for human immunodeficiency virus (HIV) was performed at 3-month intervals, using a WHO-approved rapid diagnostic test (RDT) (HIV 1 + 2 Antibody Colloidal Gold [KHB], Shanghai Kehua Bio-engineering Co., China), and a positive result was confirmed using the HIV 1/2 STAT-PAK® RDT (Chembio Diagnostics, NY, USA). At 6-month intervals, HBsAg status was assessed using a WHO-approved RDT (Determine™, Alere Inc., USA), followed by HBV DNA viral load testing using the Abbott RealTi*m*e HBV assay (Abbott Molecular, IL, USA) and from 2019 the Xpert HBV Viral Load kit (Cepheid, CA, USA). Transient elastography was performed after a minimum of 2 h fasting at 6-month intervals to assess liver fibrosis, as described previously [16, 17].

### Follow-up of patients ineligible for antiviral treatment

Patients who did not fulfil the treatment criteria were followed up with haematology and biochemistry tests every 3 months during the first year and at 6-month intervals thereafter. HBsAg status and HBV DNA viral load testing was performed every 6 months the first year, and annually thereafter. Transient elastography was performed at 6-month intervals. If patients during follow-up fulfilled the treatment criteria, then antiviral treatment was started after mandatory adherence counselling and anti-HIV testing.

### Seroconversion

If the HBsAg rapid test became negative during follow-up, a confirmatory HBsAg and anti-HBs ELISA test was performed locally (Elisys Uno, HUMAN, Wiesbaden, Germany). In the rare event of a true seroconversion (i.e. HBsAg negative and anti-HBs positive) during treatment, treatment was stopped after a consolidation period of at least 3 months.

### Adherence

Assessment of adherence to therapy was based on pill count, and adherence was calculated by dividing the total amount of tablets dispensed minus the remaining pills brought to the visit by the total number of days since the last visit. At the first visit, the patients received TDF 300 mg tablets for 30 days, and in the remaining visits, they received tablets for 90 days. Some patients living far from the clinic received tablets for 180 days.

### Diagnosis of HCC

Liver ultrasound was done at baseline and thereafter once per year in patients who started treatment, as part of the scientific evaluation of the program. More frequent monitoring in patients with cirrhosis was not possible because of travel distance and costs. If a liver lesion was detected, further evaluation with alpha-fetoprotein and radiology (computer tomography or magnetic resonance imaging) was done. The diagnosis of HCC was based on the EASL guidelines [22].

### Statistical methods

The serum aspartate aminotransferase (AST) to platelet ratio index (APRI) was calculated using the following equation: (AST [IU/L]/upper limit of normal for AST)/platelet count (10^9^/L) × 100. HBV viral load < 69 IU/mL was considered as viral suppression, whereas > 1000 IU/mL was considered as treatment failure [7, 13]. In the analysis of annual viral load trends, we accepted viral load measurements plus/minus 3 months within the first year, and minus 6/plus 3 months in the subsequent years. HBsAg loss was defined as a negative HBsAg in two consecutive samples at least 3 months apart and without later seroreversion.

Categorical variables were summarized as frequencies, whereas continuous variables were presented as median and interquartile range (IQR). Comparisons between groups were performed using Pearson chi-square tests for categorical variables and Mann–Whitney *U* tests for continuous variables. Changes over time in levels of ALT, viral load, and transient elastography were compared using Wilcoxon signed-rank tests.

The Kaplan–Meier method was used to estimate HCC-free survival, where differences between groups were assessed using the log-rank test. Cox proportional hazards regression models were used to study factors associated with HCC-free survival time and calculate hazard ratios. Univariable Cox regression analysis was performed for the following baseline variables: sex, age, ALT level, HBV DNA viral load, hepatitis B e-antigen (HBeAg), transient elastography, and cirrhosis status. Continuous variables were categorized by clinically relevant thresholds. Multicollinearity was assessed using Spearman’s correlation coefficient (*r*) with a cut-off at 0.7. Baseline variables with a *p*-value below 0.2 in univariable analyses were included in the multivariable model, using a forward stepwise method. Patients who died were right-censored at the date of death, whereas patients who developed HCC were right-censored at the date they were transferred to palliative care under the assumption that their life expectancy would be short [21]. Patients who were alive at last contact were right-censored on September 1, 2021.

The statistical analyses were performed in SPSS version 29.0 (SPSS Inc., Chicago, IL, USA). A *p*-value < 0.05 was considered significant throughout the study. The *Strengthening the Reporting of Observational studies in Epidemiology*(STROBE) statement guidelines were followed [23].

### Ethics

The study was approved by the National Research Ethics Review Committee (NRERC, Ref. No.: 3.10/829/07) in Ethiopia and by the Regional Committees for Medical and Health Research Ethics (REK Sør-Øst, Ref. No.: 2014/1146) in Norway. The study was conducted in accordance with the Declaration of Helsinki [24]. All patients gave written consent to participate in the study.

## Results

### Patient characteristics

A total of 1303 consecutive adults referred to the hospital between February 9 and December 14, 2015, were enrolled in the program, of whom 291 (22.3%) fulfilled the treatment criteria and started antiviral treatment prior to September 1, 2021. Patients eligible for treatment were more likely to be men, older, misusing alcohol, HBeAg positive, and anti-HDV positive, and having higher serum ALT, viral load, APRI-score, and liver stiffness compared to those ineligible for treatment (Table 1).

**Table 1:** Baseline characteristics of patients enrolled in a pilot treatment program for chronic hepatitis B in Ethiopia, 2015

	All patients (*n* = 1303)	Started treatment (*n* = 291)	Not started treatment (*n* = 1012)	Significance (*p*)
**Socio-demographic characteristics**				
Women	533 (40.9)	68 (23.4)	465 (45.9)	< 0.001
Age, years	31 (26–40)	33 (27–41)	30 (26–39)	0.020
18–25	286 (21.9)	60 (20.6)	226 (22.3)	0.078
26–35	549 (42.1)	108 (37.1)	441 (43.6)	
36–45	289 (22.2)	77 (26.5)	212 (20.9)	
> 45	179 (13.7)	46 (15.8)	133 (13.1)	
Alcohol misuse	45 (3.5)	21 (7.2)	24 (2.4)	< 0.001
**Laboratory tests and liver fibrosis assessment**				
ALT, IU/L^a^	25 (19–37)	36 (24–53)	23 (18–33)	< 0.001
< 40	1014 (78.9)	166 (57.0)	848 (85.3)	< 0.001
40–79	201 (15.6)	86 (29.6)	115 (11.6)	
≥ 80	70 (5.4)	39 (13.4)	31 (3.1)	
HBV viral load, IU/mL^b^	1285 (242–14,409)	34,685 (760–8,766,203)	875 (202–5522)	< 0.001
< 2000	785 (61.4)	97 (33.3)	688 (69.6)	< 0.001
2000–19,999	194 (15.2)	41 (14.1)	153 (15.5)	
≥ 20,000	300 (23.5)	153 (52.6)	147 (14.9)	
HBeAg positive^c^	142 (12.1)	85 (29.6)	57 (6.5)	< 0.001
Co-infections				
Anti-HCV positive^d^	29 (2.6)	6 (2.2)	23 (2.7)	0.660
Anti-HDV positive^e^	19 (1.5)	11 (3.9)	8 (0.8)	< 0.001
Transient elastography, kPa^f^	5.9 (4.7–8.1)	13.4 (8.4–27.4)	5.3 (4.4–6.5)	< 0.001
< 8.0	889 (74.5)	59 (21.4)	830 (90.5)	< 0.001
8.0–9.9	68 (5.7)	39 (14.1)	29 (3.2)	
≥ 10.0	236 (19.8)	178 (64.5)	58 (6.3)	
APRI-score^g^	0.24 (0.17–0.37)	0.47 (0.27–0.86)	0.21 (0.16–0.30)	< 0.001

Data are presented as number (%) or as median (interquartile range) unless otherwise noted

*Abbreviations*: *ALT* alanine aminotransferase, *APRI* aspartate aminotransferase to platelet ratio index, *HBV* hepatitis B virus, *HBeAg* hepatitis B e-antigen, *HCV* hepatitis C virus, *HDV* hepatitis D virus

^a^Serum level of ALT was missing in 18 patients, of whom all were ineligible for treatment

^b^HBV viral load was missing in 24 patients, of whom all were ineligible for treatment

^c^HBeAg status was missing in 134 patients, of whom 130 were ineligible for treatment

^d^Anti-HCV status was missing in 193 patients, of whom 170 were ineligible for treatment

^e^Anti-HDV status was missing in 36 patients, of whom 30 were ineligible for treatment

^f^Transient elastography was missing in 110 patients, of whom 95 were ineligible for treatment

^g^APRI-score was missing in 123 patients, of whom 94 were ineligible for treatment

The most common indication to start antiviral treatment was a diagnosis of cirrhosis: 105 (36.1%) patients started therapy due to decompensated cirrhosis, whereas 83 (28.5%) started due to compensated cirrhosis. The remaining patients started antiviral therapy due to significant fibrosis (*n* = 46; 15.8%), moderate/severe liver inflammation (*n* = 30; 10.3%), or a history of HCC among first-degree relatives (*n* = 7; 2.4%). A total of 20 (6.9%) patients were considered eligible even though they did not fulfil the treatment criteria at inclusion; the majority comprised of patients who had already initiated treatment through the ‘black market’ and thus could not be classified using the standard criteria due to suppressed viral load at enrolment; only one person started treatment to prevent mother-to-child transmission of HBV.

### Program loss

**Fig. 1:**
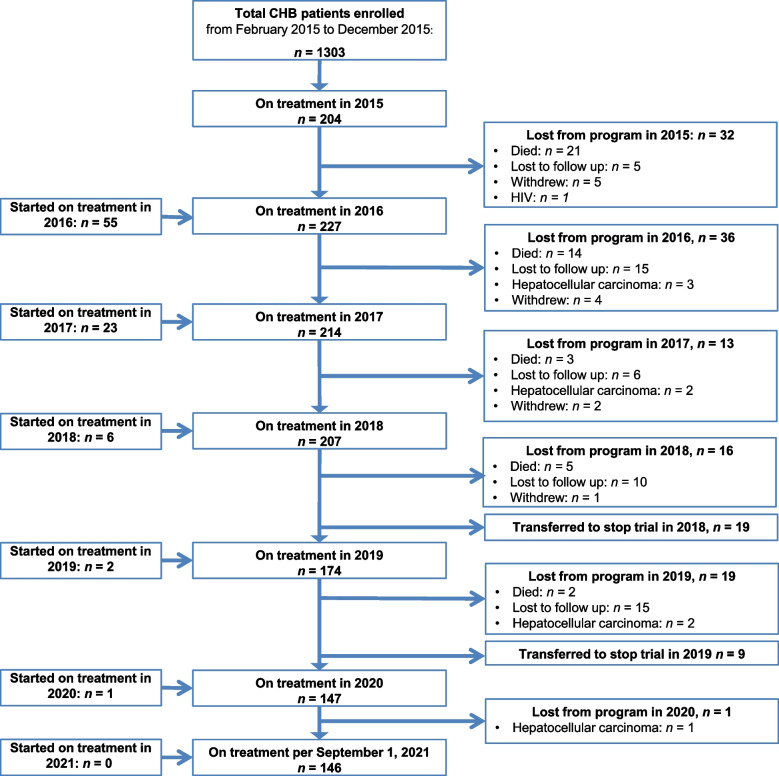
Study profile of a hepatitis B treatment program, Addis Ababa, Ethiopia, 2015–2021. Abbreviations: CHB, chronic hepatitis B

Among 291 patients who initiated antiviral therapy, 146 (50.2%) were still in care and receiving antiviral therapy after 5 years. Program loss included 51 (17.5%) patients who were lost to follow-up and 12 (4.1%) who withdrew for various reasons (Fig. 1). Another 28 (9.6%) patients were enrolled in a stop trial (ClinicalTrials.gov identifier: NCT03681132) after approximately 3 years of treatment and therefore stopped taking TDF although they were still in care. Nine (3.1%) patients were transferred out, of whom eight were diagnosed with HCC and referred to palliative care, and one was diagnosed with HIV and transferred to HIV care. This latter person had a negative HIV RDT at baseline and a positive HIV RDT on her 3-month follow-up appointment; however, on re-testing with an enzyme-linked immunosorbent assay, the baseline sample turned out to be positive so this person was already infected with HIV at inclusion.

### Mortality

A total of 45 (15.5%) patients died within the initial 5-year course of antiviral treatment, of whom, 40 (88.9%) had decompensated cirrhosis at baseline. The majority (*n* = 29; 64.4%) died within the first year of treatment. Among patients with decompensated cirrhosis, the estimated proportion who were dead or diagnosed with incurable HCC was 28.7% after 1 year of antiviral therapy; however, the mortality decreased in the subsequent years: the estimated proportion who were dead or had HCC was 40.3% after 3 years and 45.8% after 5 years.

**Fig. 2:**
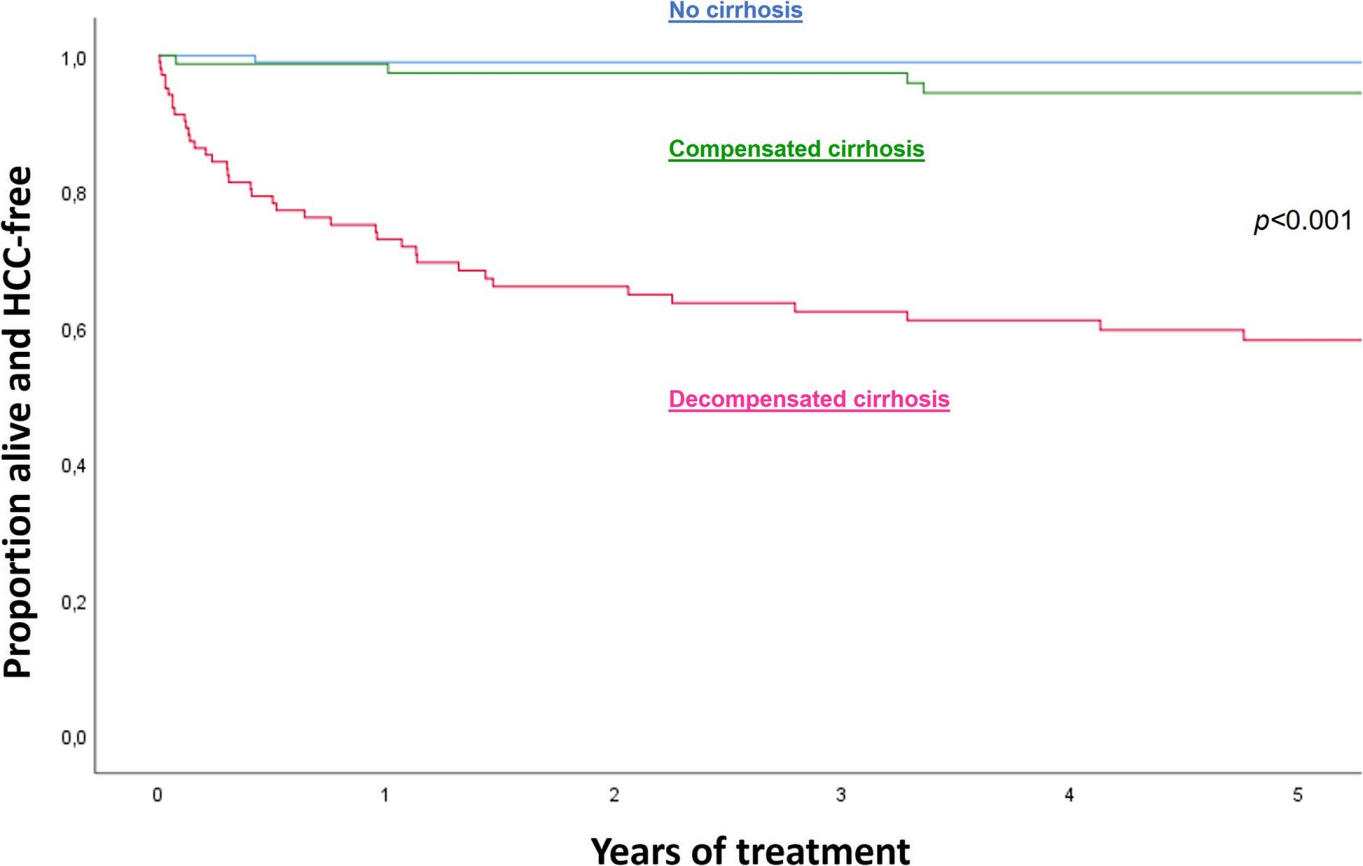
HCC-free survival in patients on TDF therapy, Addis Ababa, Ethiopia. Abbreviations: HCC, hepatocellular carcinoma; TDF, tenofovir disoproxil fumarate

Figure 2 illustrates the 5-year HCC-free survival among patients on treatment, based on the presence of cirrhosis at baseline. The estimated 5-year survival was 99.0% in patients without cirrhosis, compared to 88.8% in patients with compensated cirrhosis, and 54.2% in patients with decompensated cirrhosis (*p* < 0.001) (Fig. 2; Additional file 1: Figure S1).

In multivariable Cox regression analysis, the risk of death/HCC was substantially higher in patients with decompensated cirrhosis at baseline (adjusted hazard ratio [AHR] 44.6, 95% confidence interval [CI] 6.1–328.1; *p* < 0.001) and in patients older than 40 years (AHR 3.7, 95% CI 1.6–8.5; *p* = 0.002) (Table 2). Transient elastography was not included in the adjusted model as it was strongly correlated with the cirrhosis variable (*r* = 0.779, *p* < 0.001).

**Table 2:** Baseline risk factors for death or hepatocellular carcinoma among HBV-infected patients who started antiviral therapy

	Total (*n* = 291)	Deceased^a^ (*n* = 53)	Crude hazard ratio (95% CI)	Significance (*p*)	Adjusted hazard ratio (95% CI)	Significance (*p*)
**Baseline variables**						
Sex (female *vs.* male)						
Male	223 (76.6)	44 (83.0)	1			
Female	68 (23.4)	9 (17.0)	0.6 (0.3–1.3)	0.237	NA	NA
Age (*vs.* < 30 years)						
18–29	98 (33.7)	7 (13.2)	1		1	
30–39	103 (35.4)	16 (30.2)	2.3 (0.9–5.5)	0.069	1.5 (0.6–3.6)	0.404
≥ 40	90 (30.9)	30 (56.6)	5.1 (2.2–11.5)	< 0.001	3.7 (1.6–8.5)	0.002
Alcohol misuse (*vs*. no misuse)						
No	270 (92.8)	49 (92.5)	1			
Yes	21 (7.2)	4 (7.5)	1.1 (0.4–3.0)	0.860	NA	NA
ALT (*vs.* < 40 IU/L)						
< 40	166 (57.0)	31 (58.5)	1			
40–79	86 (29.6)	14 (26.4)	1.0 (0.5–1.8)	0.896	NA	NA
≥ 80	39 (13.4)	8 (15.1)	1.2 (0.6–2.6)	0.647	NA	NA
HBV DNA viral load (*vs.* < 2000 IU/mL)						
< 2000	91 (31.3)	21 (39.6)	1		1	
2000–20,000	47 (16.2)	5 (9.4)	0.5 (0.2–1.3)	0.165	0.9 (0.3–2.4)	0.795
> 20,000	153 (52.6)	27 (50.9)	0.8 (0.5–1.4)	0.456	1.2 (0.7–2.1)	0.626
HBeAg status (positive *vs*. negative)^b^						
Negative	202 (70.4)	40 (75.5)	1			
Positive	85 (29.6)	13 (24.5)	0.7 (0.4–1.4)	0.316	NA	NA
Transient elastography (*vs.* < 8.0 kPa)^c^						
< 8.0	59 (21.4)	1 (2.0)	1			
8.0–9.9	39 (14.1)	1 (2.0)	1.6 (0.1–25.3)	0.745	NA^d^	NA^d^
≥ 10.0	178 (64.5)	47 (95.9)	16.6 (2.3–120.5)	0.005	NA^d^	NA^d^
Cirrhosis status (*vs.* no cirrhosis):						
No cirrhosis	103 (35.4)	1 (1.9)	1		1	
Compensated cirrhosis	83 (28.5)	8 (15.1)	9.0 (1.1–72.3)	0.038	7.2 (0.9–58.6)	0.065
Decompensated cirrhosis	105 (36.1)	44 (83.0)	52.2 (7.2–379.3)	< 0.001	44.6 (6.1–328.1)	< 0.001

Data are presented as number (%)

^a^Eight patients developed hepatocellular carcinoma and were registered as deceased in the survival analysis due to the poor prognosis in sub-Saharan Africa [19]

^b^HBeAg status was missing in four patients, of whom none died or developed hepatocellular carcinoma

^c^Transient elastography was missing in 15 patients, of whom three died and one developed hepatocellular carcinoma

^d^Transient elastography could not be included in the adjusted model because of a strong correlation with one of the other variables

### Treatment response

Among the 120 individuals who completed 5 years of antiviral treatment by September 1, 2021, the median ALT at baseline was 36 IU/L (IQR 22–49) and did not change significantly throughout the 5-year course of TDF therapy: median (IQR) ALT after 1 year was 34 IU/L (26–39) (*p* = 0.518), 35 IU/L (26–48) (*p* = 0.867) after 3 years, and 36 IU/L (25–47) (*p* = 0.336) after 5 years.

**Fig. 3:**
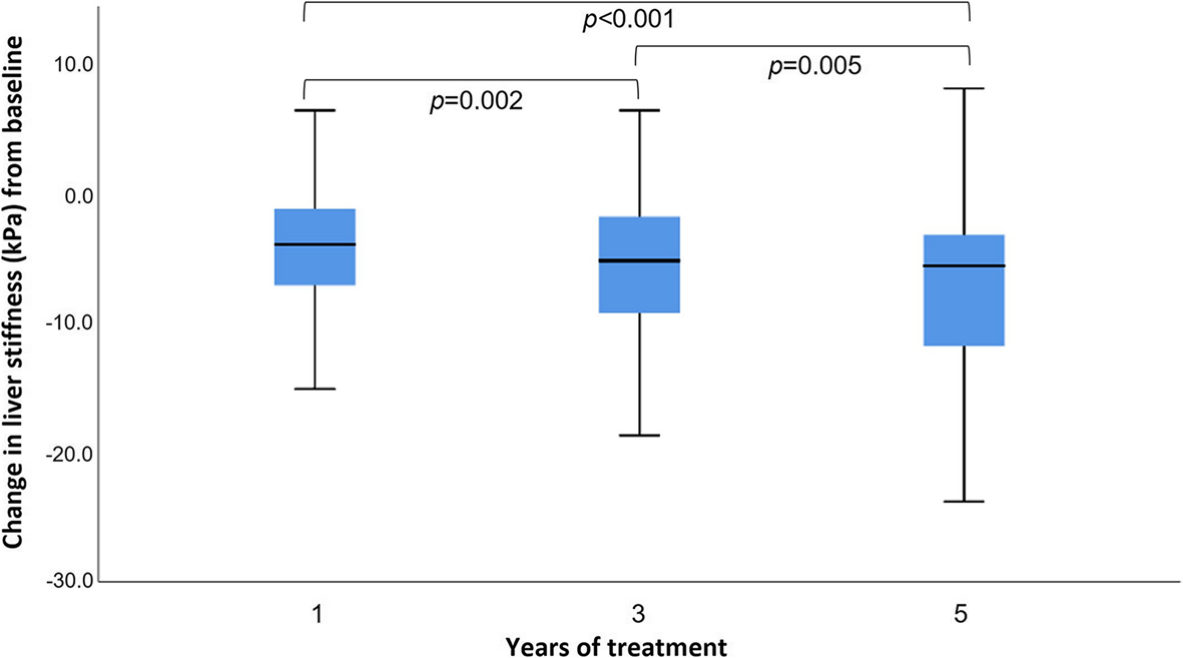
Change in liver stiffness among patients who completed 5 years of TDF therapy. Abbreviations: TDF, tenofovir disoproxil fumarate. *P* values refer to comparisons of liver stiffness changes after 1, 3, and 5 years

Median (IQR) liver stiffness at baseline was 13.8 kPa (9.9–22.2) and declined significantly compared to baseline throughout the 5-year course of TDF therapy: median (IQR) liver stiffness was 10.1 kPa (6.6–15.2) (*p* < 0.001) after 1 year, 8.4 kPa (5.8–14.8) (*p* < 0.001) after 3 years, and 7.2 kPa (5.3–12.8) (*p* < 0.001) after 5 years. A total of 74 patients on antiviral treatment without ascites at baseline had repeated liver stiffness measurements up to 5 years; the median (IQR) change in liver stiffness compared to baseline was − 4.0 kPa (− 7.2 to − 1.1) after 1 year, − 5.2 kPa (− 9.4 to − 1.7) after 3 years, and − 5.6 kPa (− 11.8 to −  − 3.2) after 5 years (Fig. 3).

**Fig. 4:**
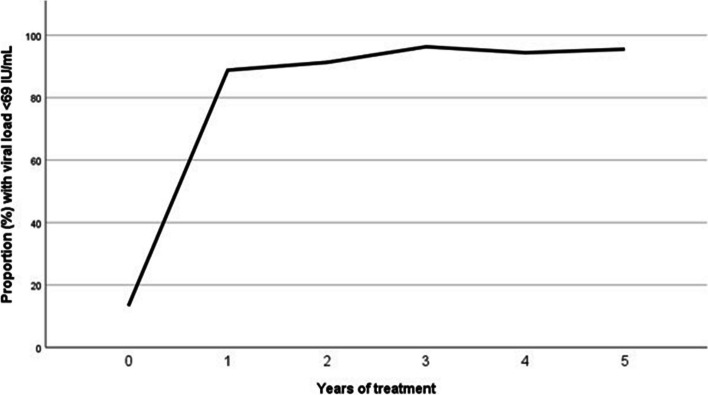
Virological response to TDF therapy. Abbreviations: TDF, tenofovir disoproxil fumarate. Hepatitis B viral load of less than 69 IU/mL was considered as viral suppression

Out of 108 patients who had HBV viral load results available after 4 years of treatment, 102 (94.4%) had suppressed viraemia (i.e. viral load < 69 IU/mL), whereas only four (3.7%) had treatment failure (i.e. viral load > 1000 IU/mL). Due to the pandemic lockdown, viral load results were available only in 44 (36.7%) individuals who had completed 5 years of treatment, of whom 42 (95.5%) had maintained viral suppression (Fig. 4).

Among the 291 individuals who started antiviral treatment, only two (0.7%) experienced HBsAg loss, both of which occurred within 12 months after treatment initiation; one of the two also developed anti-HBs.

### Adherence

Pharmacy refill data was available for 290 out of 291 patients who started antiviral treatment. Overall, 164 (56.6%) patients had excellent adherence to therapy (i.e. took ≥ 95% of their pills); 89 (30.7%) patients had good adherence (i.e. took 80.0–94.9% of their pills); and, 37 (12.8%) patients had poor adherence (i.e. took < 80.0% of their pills). There were no significant differences in adherence between men and women, age groups, or rural *vs.* urban residency. For patients who completed 5 years of therapy, the overall adherence to therapy was 95.4% and there was no decline in adherence over time.

### Adverse events

In general, TDF therapy was well tolerated. One patient discontinued treatment due to progressive renal impairment during the first year of antiviral therapy. This individual was under treatment of several comorbidities, including hypertension (treated with angiotensin-converting enzyme inhibitor), cardiomyopathy (using diuretics and statins), type 2 diabetes mellitus (using three different anti-diabetic drugs), and alcohol abuse, and thus, the underlying cause of renal failure was probably multifactorial.

## Discussion

This study reports long-term patient outcomes in one of the first large-scale treatment programs for CHB in sub-Saharan Africa. We found that the overall mortality was high (15.5%) among patients who started antiviral therapy, with most deaths occurring among those with decompensated cirrhosis at baseline. This underscores the importance of improved access to diagnosis and treatment in sub-Saharan Africa, so antiviral therapy can be initiated before irreversible liver injury has occurred. Notably, the estimated 5-year HCC-free survival was excellent (99%) among individuals who initiated TDF therapy without evidence of cirrhosis, compared to 89% among patients with compensated cirrhosis, and 54% in patients with decompensated cirrhosis. As our group has shown earlier, the current WHO treatment guidelines for CHB mainly identify patients with cirrhosis as treatment eligible [17]. The present study is a strong argument for more inclusive treatment criteria, where the aim must be to initiate antiviral therapy before the advent of cirrhosis.

On the other hand, the benefit of antiviral therapy was evident even in patients with decompensated cirrhosis. The mortality in this group was high during the initial year of treatment, but in subsequent years, the mortality decreased significantly and more than half of the patients were still alive without HCC after 5 years. Our results compare well with a study of 2682 decompensated CHB patients from Korea, where the estimated 5-year mortality was 32.6% and cumulative 5-year incidence of HCC was 24.1% [25]. As in our study, the authors found that both mortality and HCC incidence were drastically reduced after the initial 1–2 years of antiviral therapy; indeed, the annual HCC incidence appeared to be quite similar (around 2%) in patients with compensated and decompensated cirrhosis after 2 years of therapy.

Reversal of fibrosis was an important finding in our study. The liver stiffness was significantly reduced during the 5-year course of antiviral therapy, and the beneficial effect on liver stiffness appeared to continue even after the initial years of therapy. This is in line with results from Marcellin et al., [7] who found reversal of liver fibrosis in 51% of CHB patients after 5 years on TDF therapy. Our findings suggest that the beneficial effect of antiviral therapy on the liver is sustained over at least 5 years, which has not previously been shown in sub-Saharan Africa.

We found that long-term TDF therapy is safe and effective in this setting, with suppression of HBV DNA in the vast majority of patients over a time period of 5 years. Viral suppression was maintained in around 95% of patients receiving TDF therapy, and treatment failure was consistently low (< 5%) throughout the 5-year observation time. Our results are comparable to studies in high-income settings; indeed, a 10-year follow-up study from resource-rich settings showed viral suppression in 98% of patients on TDF therapy with no development of drug resistance [13].

HCC is thought to occur in HBV-infected patients even without cirrhosis [2], yet in our cohort, all individuals who developed HCC had cirrhosis at baseline. Previous studies have shown that HBV-related HCC in African patients is diagnosed late and associated with a grim prognosis [21]. In Ethiopia, like most countries in sub-Saharan Africa, there are few surgical centres that offer liver resection and no liver transplant facilities; hence, curative treatment for HCC is rarely possible. Prevention of HCC through HBV vaccination and expanded access to HBV treatment, therefore, must be a top priority [26].

Overall, adherence to TDF therapy was high. A high percentage of patients had excellent or good adherence to therapy, which is comparable or better than reports from hepatitis B cohorts in high-income countries [27, 28]. There were no significant differences in adherence levels based on sex or age, which differs from other studies in which younger patients had lower adherence [28]. Of note, adherence to therapy did not drop over time; it remained high for up to 5 years.

Program loss occurred throughout the 5-year follow-up and 24.7% were either lost to follow-up, transferred out, or withdrawn from the program 5 years after enrolment. Although this drop-out rate was relatively high, it is similar to studies from high-income settings, where around 75% remain in care after 3 to 7 years of follow-up [7, 9–11].

Our study had certain limitations. First, the cohort was established at a referral hospital, and it is likely that there was a selection bias towards patients with more advanced liver disease. Hence, the high mortality observed in our study is probably not representative of all CHB treatment programs in the region. Second, the potential role of concurrent metabolic dysfunction-associated fatty liver disease (MAFLD) could not be assessed as controlled attenuation parameter (CAP) was unavailable and abdominal ultrasound was only performed in patients who started TDF therapy. Although body mass index was calculated at baseline, it has limited value as an indicator of metabolic dysfunction in patients with ascites. Third, during the third and fourth years, around 10% of patients on treatment were transferred to a stop trial. These patients were selected since they had sustained viral load suppression and no cirrhosis at inclusion. If this group was retained on treatment, we would have observed an even better treatment response in terms of viral suppression and adherence to therapy. Fourth, antiviral treatment and care within the program was provided free of charge which may have inflated the level of adherence and retention in care. Fifth, our study was not powered to assess the effect of adherence on outcomes. Finally, estimating adherence using pill count as the measurement tool has its limitations. Although pill count might provide more correct adherence estimates compared to methods like re-call and questionnaires, pills can be lost or misused, and patients might forget to bring forth all pills to their provider, resulting in over-estimation of actual adherence [29].

## Conclusions

In a large hepatitis B treatment program in Ethiopia, we found that CHB therapy was feasible and effective in a resource-limited setting. We found beneficial long-term clinical effects with reduction in liver stiffness and maintained virological suppression throughout the 5 years of follow-up. Five-year HCC-free survival was excellent in patients without cirrhosis at enrolment, but only roughly 50% in patients with decompensated cirrhosis, underscoring the importance of earlier diagnosis and initiation of antiviral treatment — before the onset of cirrhosis. Our study advocates for further scale-up and expansion of CHB treatment services in sub-Saharan Africa, which will be vital to reach the WHO elimination goals by 2030.

### Supplementary Information

**Additional file 1: Figure S1.** Kaplan-Meier plot and patients-at-risk table showing HCC-free survival in patients on TDF therapy, Addis Ababa, Ethiopia.

## Data Availability

The datasets used and/or analysed during the current study are available from the corresponding author on reasonable request.

## Abbreviations

AHR: Adjusted hazard ratio
ALT: Alanine aminotransferase
APRI: Aspartate aminotransferase to platelet ratio index
AST: Aspartate aminotransferase
CHB: Chronic hepatitis B
CI: Confidence interval
EASL: European Association for the Study of the Liver
HBeAg: Hepatitis B e-antigen
HBsAg: Hepatitis B surface antigen
HBV: Hepatitis B virus
HCC: Hepatocellular carcinoma
HCV: Hepatitis C virus
HDV: Hepatitis D virus
HIV: Human immunodeficiency virus
IQR: Interquartile range
RDT: Rapid diagnostic test
STROBE: Strengthening the Reporting of Observational studies in Epidemiology
TDF: Tenofovir disoproxil fumarate
WHO: World Health Organization
