# Supplementary material for: Five-year results of a treatment program for chronic hepatitis B in Ethiopia
Source: BMC Med. 2023 Sep 29;21:373. doi: 10.1186/s12916-023-03082-4 (PMC10543851; doi:10.1186/s12916-023-03082-4)
Supplement: Supplementary file 1 — Additional file 1: Figure S1. Kaplan-Meier plot and patients-at-risk table showing HCC-free survival in patients on TDF therapy, Addis Ababa, Ethiopia. [file 12916_2023_3082_MOESM1_ESM.docx]

**Additional file 1: Figure S1.** Kaplan-Meier plot and patients-at-risk table showing HCC-free survival in patients on TDF therapy, Addis Ababa, Ethiopia

**Abbreviations: HCC**, hepatocellular carcinoma; **TDF**, tenofovir disoproxil fumarate.

**
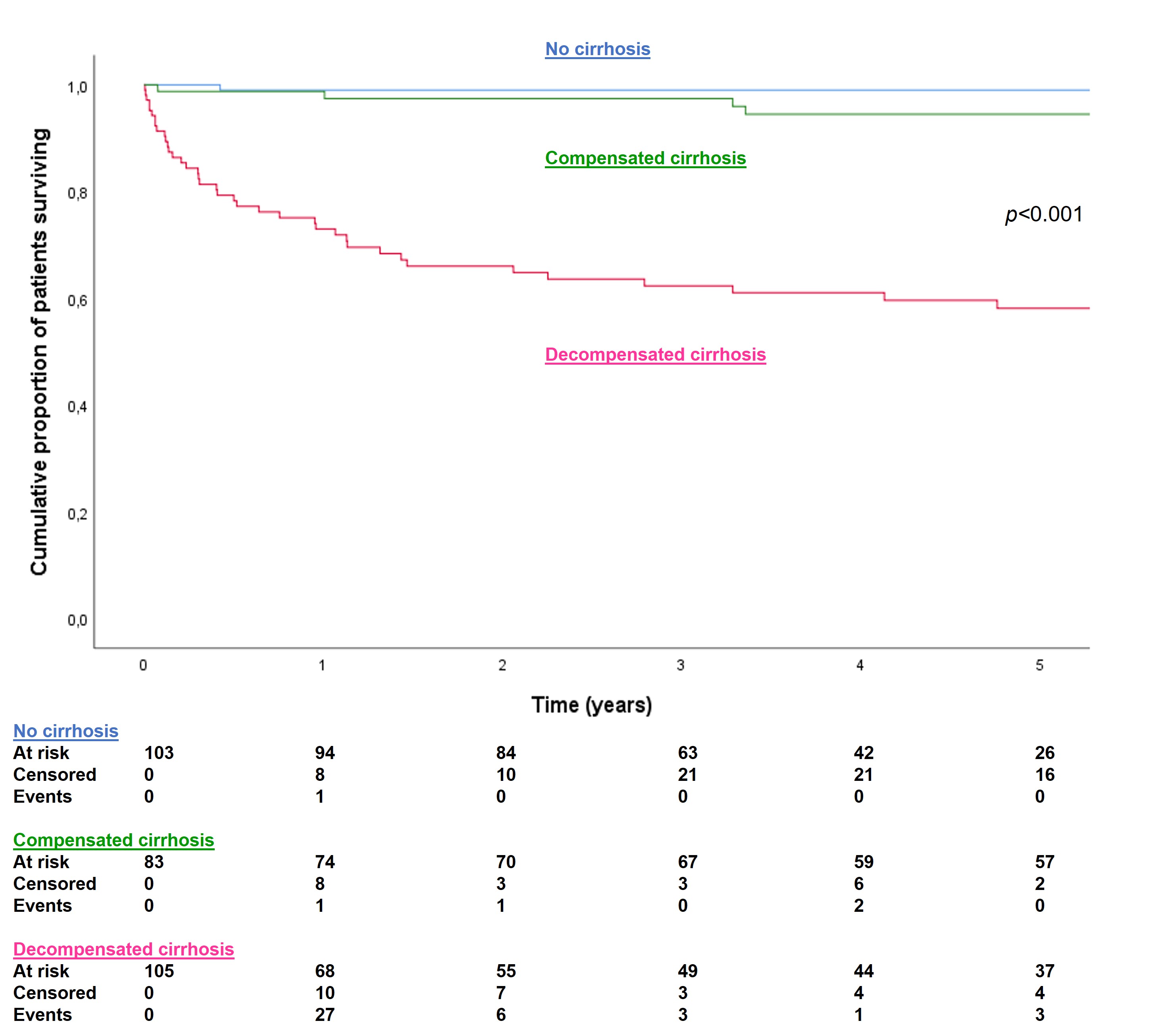
**
